# Photoreceptor assessment in focal laser-treated central serous chorioretinopathy using adaptive optics and fundus autofluorescence

**DOI:** 10.1097/MD.0000000000019536

**Published:** 2020-04-10

**Authors:** Radu Ochinciuc, Uliana Ochinciuc, Horia T. Stanca, Ramona Barac, Diana Darabus, Marius Şuţă, Florian Baltă, Marian Burcea

**Affiliations:** a Department of Ophthalmology, “Victor Babes” University of Medicine and Pharmacy, Timisoara; b Department of Ophthalmology, “Dr. Carol Davila” Central Military Emergency University Hospital; c Department of Ophthalmology, “Carol Davila” University of Medicine and Pharmacy, Bucharest, Romania.

**Keywords:** adaptive optics, cone mosaic, fundus autofluorescence, laser-treated central serous chorioretinopathy, photoreceptor density

## Abstract

This study analyzed cone density, cone mosaic, and fundus autofluorescence (FAF) images in patients with focal laser-treated central serous chorioretinopathy (CSC).

Observational case series.

Forty-two eyes of 21 patients with unilateral treated CSC and bilateral best-corrected visual acuity of 1.0 (decimal fraction) were included. FAF and cone mosaic images were obtained in all patients with an adaptive optics fundus camera. Densities were recorded at 20 points throughout the macula, and choroidal thicknesses were measured.

Mean choroidal thicknesses were 419.95 ± 110.33 μm in normal eyes, 459.09 ± 90.07 μm in eyes with active CSC, and 438.61 ± 107.57 μm in treated eyes. The highest density of cones in healthy eyes was 38146 cones/mm^2^, with a 5.66-μm intercellular space (IS), at 700 μm temporal to the center. In eyes with treated CSC, the highest density was 32749 cones/mm^2^, with a 6.13-μm IS, at 500 μm nasal to the center. In all quadrants, median values of maximum cone density were significantly higher in healthy eyes (*P* = .02, *P* = .003, *P* = .0001, and *P* = .001). Three types of lesions were identified on FAF and were correlated with those on cone mosaic images. Strong correlations were detected between the presence of hypoautofluorescent lesions on the first FAF image and a greater difference between maximum values of photoreceptor density (*r* ^2^ = 0.46, *P* = .03), as well as between the presence of hypoautofluorescent lesions and the duration of pathology (*r* ^2^ = 0.68, *P* < .001).

The presence of hypoautofluorescent lesions and the duration of pathology were negative prognostic factors in CSC. Laser treatment could prevent photoreceptor loss.

## 1 Introduction

Central serous chorioretinopathy (CSC) is the fourth most common type of maculopathy, characterized by serous detachment of the neurosensory retina, sometimes associated with retinal pigment epithelium (RPE) detachment.^[1]^ Recent pachychoroid studies with spectral domain optical coherence tomography (SD-OCT) and indocyanine green angiography suggest that hyperperfusion and hyperpermeability of the choroid are part of the pathophysiological mechanism of CSC.^2^,^3^ Fundus autofluorescence (FAF) imaging allows a detailed appreciation of structural changes in the photoreceptor and RPE layers^[4]^ and permits a thorough assessment of their functioning as well.^2^,^5^

Adaptive optics (AO) is an innovative technology that allows for the acquisition of retinal images of a resolution up to 2 μm.^6^,^7^ It is one of the few non-invasive modalities that provide sufficient information on very fine retinal structures.^[8]^ A decrease in photoreceptor density, as well as a few cone mosaic patterns, were found using this imaging method in CSC studies.^9^,^10^,^11^

This study was performed to analyze AO and FAF results, to determine whether laser therapy is advantageous in CSC treatment. The study investigated potential correlations between FAF and high-resolution AO images; it also assessed some parameters of CSC with negative impacts on photoreceptors.

## 2 Methods

This was an observational case study of 21 patients from the Retina clinic, with unilateral laser-treated CSC during the period January, 2016 to February, 2019. The study was retrospectively approved by the Institutional Review Board and the Ethics Committee of Ponderas Academic Hospital. All procedures conformed to the tenets of the World Medical Association's Declaration of Helsinki and written informed consent was obtained from all patients.

## 3 Patients

This study included 21 eyes with treated CSC and 21 fellow, control eyes with no disease. Inclusion criteria were: 1 eye with treated CSC and no subretinal fluid (SRF) for at least 6 months, and the fellow eye without any ocular pathologies; best-corrected visual acuity (BCVA) for both eyes = 1.0 (decimal fraction; 6/6 Snellen VA); CSC affecting the center of the macula.

Diagnosis of CSC was based on the results of slit-lamp biomicroscopy examination, OCT, FAF, and fluorescein angiography. After the leakage point was localized, focal laser photocoagulation with 532 nm laser therapy was performed on all patients. All patients who met the inclusion criteria were examined with the AO Retinal Camera rtx1 (Imagine Eyes, France). For all patients, the duration of the pathology was calculated in months beginning with the onset of symptoms until complete SRF resorption. The duration of SRF resorption was measured in months from the day laser therapy was performed to the day disappearance of SRF was observed on OCT images.

## 4 Adaptive optics image acquisition and analyses

AO Retinal Camera rtx1 is a fully automated, low-noise, charge-coupled device, with near infrared LED (850 nm), 1600 μm depth focusing range, 250 line pairs per millimeter resolution, and 1.1 μm pixel pitch on the fundus.^[12]^

For each eye, a high-resolution image of the fovea's center and 8 more images of the area around it were captured by moving the fixation point. In all cases, the cone mosaic appeared at 70 μm depth of focus. The AOimage software (Imagine Eyes, France) was used to obtain the images. Image alignment and multi-image mosaics were obtained with the help of the i2k retina software (Imagine Eyes, France). After image processing, we had pictures of an 8° × 8° area of the retina with fovea in the center.

Measurements of cone density were performed automatically using the AOdetect mosaic retina software (Imagine Eyes, France). We ensured that the fovea was properly centered in the image for each case. The measured area was a square with a surface area of 100 μm^2^ that was set manually by marking the distance from the center of the fovea to the area to be analyzed. Measurements were made from the center of the fovea up to 1500 μm from it (15 measurements of 100 μm^2^ area) in all 4 quadrants (superior, inferior, nasal, and temporal), thus obtaining 61 measurements (including the center) for each eye. For this study, we used densities from 20 points, at 400, 500, 600, 700, and 800 μm from the center to all 4 directions. Since the area with maximum density was not at any of these points in some cases, it was noted separately for each eye, as well as the distance at which it was found and the size of the intercellular space (IS) in that area. The distinction between the densities of affected and healthy eyes was determined by calculating the ratio between them.

For measurements of cone density, the AO Detect mosaic software (Imagine Eyes, Orsay, France) required the axial length (AL) of the eye. It was measured with the Aladdin HW 3.0 Biometer with Corneal Topography from Topcon.

## 5 Optical coherence tomography: fundus autofluorescence, pachychoroid, and subretinal fluid measurements

Swept Source DRI OCT Triton plus (TOPCON, Tokyo, Japan) was used in all cases. FAF imaging and 6-mm radial scans were performed for all patients at each visit. The measurements of all OCT variables were made from the horizontal scan line crossing the foveal center. Outer retinal thickness (ORT)^[13]^ was measured as the distance between the outer border of the external limiting membrane and the inner border of the RPE. It included the thickness of the interdigitation zone (IZ),^[14]^ ellipsoid zone, and the SRF. Choroidal thickness (ChT) was measured between the outer border of the RPE and the outer border of choroid. The highest thickness was considered for the measurement of ORT. ChT was measured in both eyes, in the subfoveal area. In eyes with treated CSC, the ChT was measured on OCT scans for eyes with active CSC and eyes with resolved CSC as well.

## 6 Statistical analysis

Distribution of variables was verified using the Shapiro-Wilk test. Mann–Whitney *U* test was used to compare photoreceptor densities and ChT. Spearman rank correlation coefficient was calculated to estimate correlations between photoreceptor densities and some disease parameters that were found on OCT. Microsoft Excel 2010 (Microsoft, United States) and IBM SPSS Statistics Base 22.0 (IBM, United States) software were used for all statistical evaluations. A *P* value <.05 was considered statistically significant. Best-corrected visual acuity was measured using the ETDRS chart on CC-100XP test chart (TOPCON, Tokyo, Japan) and was expressed in decimal fraction.

## 7 Results

This study included 42 eyes from 21 patients (13 men, 8 women), with a mean age (mean ± standard deviation) of 39.52 ± 9.13 years (range, 27–61 years). Mean BCVA at first presentation was 0.75 ± 0.21 (range, 0.1–1; 6/60–6/6 Snellen) and the mean duration of pathology was 10.66 ± 10.41 months (range, 1–32 months). The duration of SRF resorption was 1 month in 14 cases (66.66%), with a mean value of 1.6 months (range, 1–6 months). The mean AL was 23.4 ± 0.76 mm in healthy eyes and 23.39 ± 0.75 mm in eyes with CSC. All variables calculated using OCT images are outlined in Table 1.

**Table 1:**
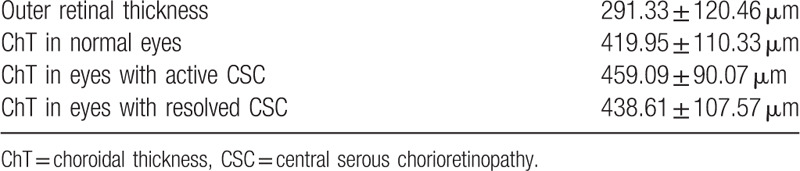
Mean values of all variables calculated from optical coherence tomography images.

In most cases, the choroid was thinner in healthy eyes than in eyes with active CSC (14 cases [66.66%], *P* = .2). Regarding the comparison of eyes with CSC before and after laser therapy, 6 months after SRF resorption, the choroid was observed to be thinner in 15 eyes with resolved CSC (71.42%, *P* = .5).

**Figure 1:**
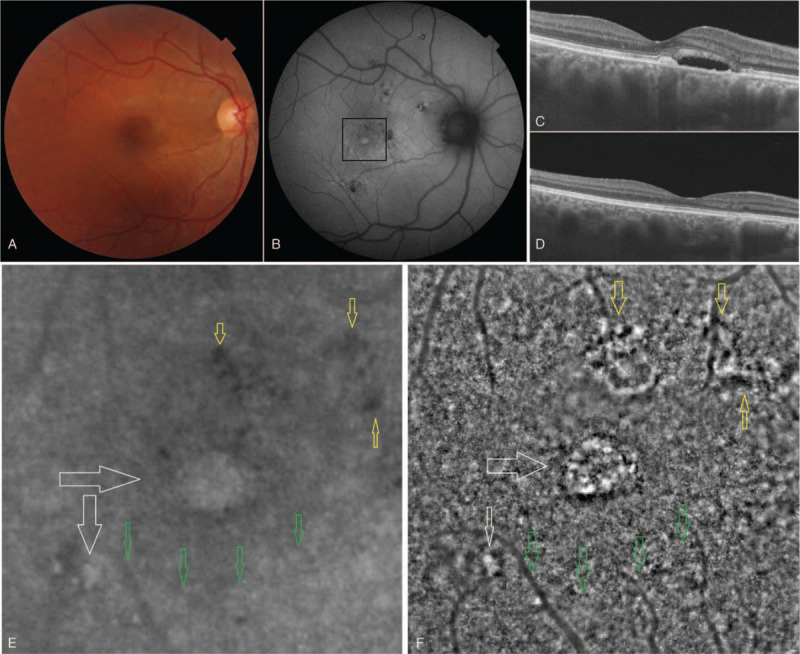
Central serous chorioretinopathy sequelae visible on the cone mosaic. A. Color fundus image. B. Fundus autofluorescence image. C–D. Optical coherence tomography scan of the same case during active and resolved central serous chorioretinopathy. E. Magnified view of the area outlined in black in (B). F. High-resolution image of the cone mosaic, 8° × 8° section with the fovea in the center, which corresponds to the area in (E) showing predominantly black lesions of hypoautofluorescence zones (yellow arrows); well-delimited central white lesions surrounded by black lesions. Below and to the left, smaller lesions that correspond to hyperautofluorescence zones (white arrows); blurred area of cone mosaic showing patchy increased autofluorescence (green arrows).

On FAF images, 3 types of lesions were identified: intense hyperautofluorescent (Hyper-AF) precipitates, patchy increased autofluorescence (AF), and hypoautofluorescent (hypo-AF) lesions.^[4]^ In all eyes with resolved CSC, Hyper-AF lesions were present and few of the eyes also had patchy increased AF and associated hypo-AF injuries. FAF and AO images were correlated (Fig. 1). Intense Hyper-AF precipitates appeared as intense white regions with poorly visible cones observed through them, surrounded by or adjacent to small dark regions without cones. Patchy increased AF appeared as a well-defined, blurred, hazy area of the cone mosaic. Hypo-AF lesions appeared as intense dark regions without cones. In most cases, the mosaic had other areas with altered cone distribution that had no correlations on the FAF images.

**Figure 2:**
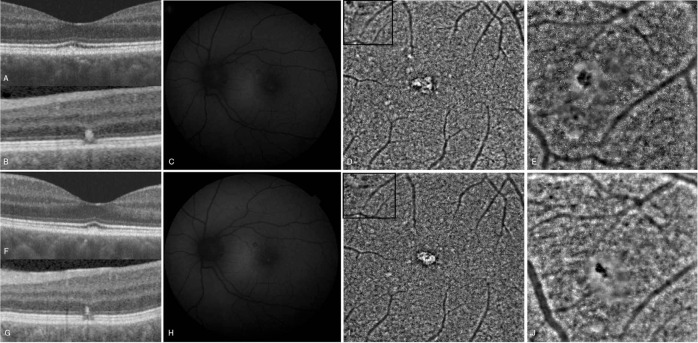
Evolution of central serous chorioretinopathy scar and laser photocoagulation area on fundus autofluorescence and adaptive optics high-resolution images. A, B, F, G. Optical coherence tomography (OCT) images obtained 4 months after laser treatment, showing hyper-reflective material under the interdigitation zone in the fovea (A) and interruption of the interdigitation zone and the ellipsoid zone, with alteration of the inner layers in photocoagulated area (B); OCT image obtained after 10 months showing a decrease of the material (F) with a slight regeneration of the outer layers (G). C, H. Fundus autofluorescence images after 4 months showing the presence of a hyperautofluorescent zone in the center of the fovea and many small encircling lesions, with a hyperautofluorescent middle surrounded by a hypo-autofluorescent laser scar (C); all lesions decreased in size and number over 10 months, with the exception of the laser scar that showed an increase in hyperautofluorescence and a decrease of hypoautofluorescence, indicating an active metabolic process (H). D, I. High-resolution image of the cone mosaic, an 8° × 8° section with the fovea in the center, which shows a decrease in the number and size of white and black areas on the image after 10 months (I), compared with the image obtained after 4 months (D). With the exception of the white and black lesions scattered in the center, the remaining mosaic appears normal. E, J. Magnified view of the laser scar outlined in black in (D, I); black lesions appear to decrease in size at 10 months (J), compared with the image at 4 months (E). In the image at 10 months, the blurred area around the black lesion is larger, and the remaining portion of the image appears similar to a normal mosaic (J).

Due to the presence of the pre-existing macular lesions, it was difficult to make correlations of the laser scar appearance in all patients. However, black lesions predominated in the center of the laser lesion and a blurry area or white spots were observed around it. On FAF, it appeared Hyper-AF in the center and hypo-AF around the center, or generally hypo-AF (Fig. 2).

Shapiro-Wilk test results of the affected eyes showed an abnormal distribution at all distances from the center of the fovea in the affected eyes. In normal eyes, except for 600 μm (*w* = 0.96, *P* = .033, 95% confidence interval [0.9704–1.0000]) and 700 μm (*w* = 0.96, *P* = .014) from the center, all cone densities had a Gaussian distribution. The highest cone density detected in healthy eyes was 37,983 cones/mm^2^, with a 5.66 μm IS, found at 700 μm from the center, in the temporal quadrant of an eye with a 22.02 mm AL; in eyes with resolved CSC, respective values were 32,749 cones/mm^2^, 6.13 μm IS, 500 μm from the center, in the nasal quadrant of an eye with a 21.66 mm AL (Table 2). The cones dispersion in these points was 8.8% in the healthy eye, and 13.1% in the affected one.

**Table 2:**
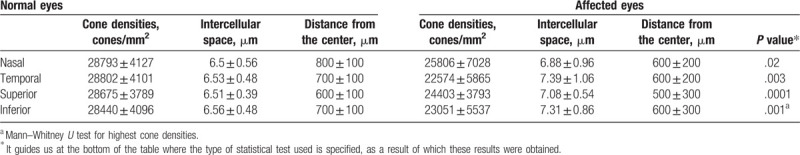
Highest cone densities, their intercellular spaces, and the distance from the center where they were found (median ± interquartile range) in normal and affected eyes.

**Figure 3:**
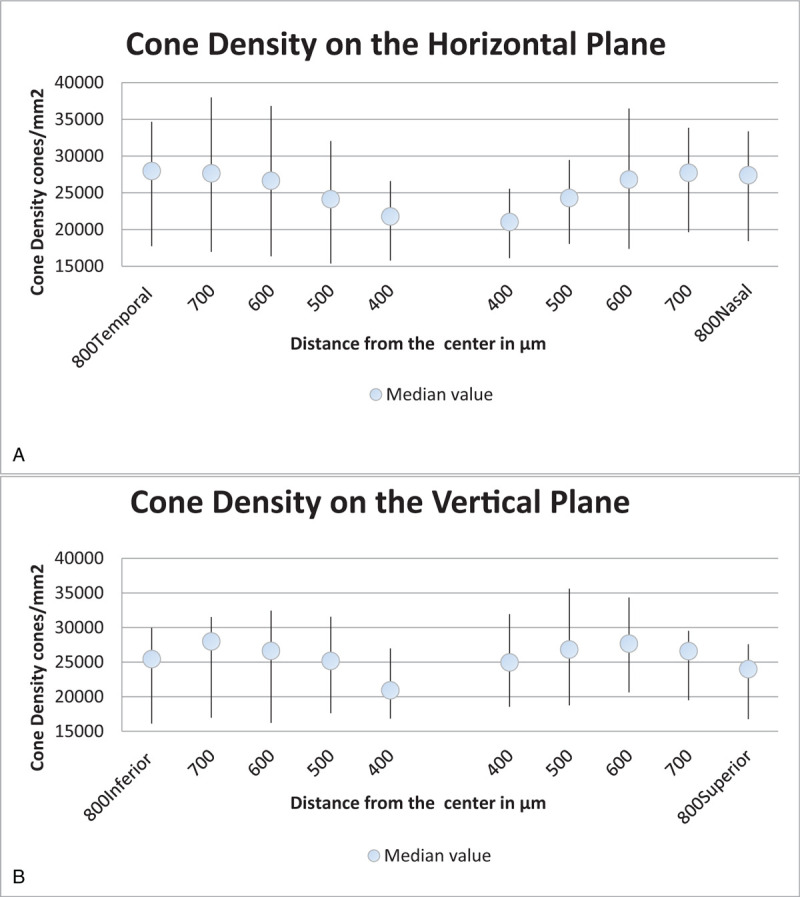
Cone density in healthy eyes. (A) Cone density on the horizontal plane. (B) Cone density on the vertical plane.

**Figure 4:**
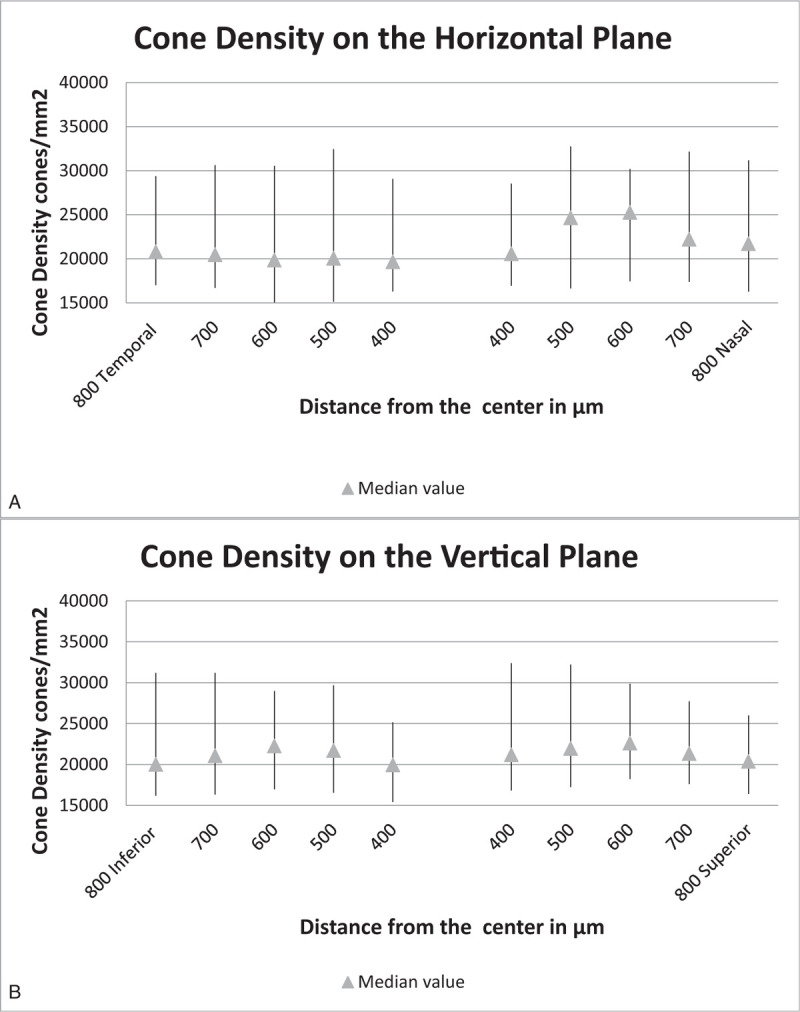
Cone density in affected eyes. (A) Cone density on the horizontal plane. (B) Cone density on the vertical plane.

**Figure 5:**
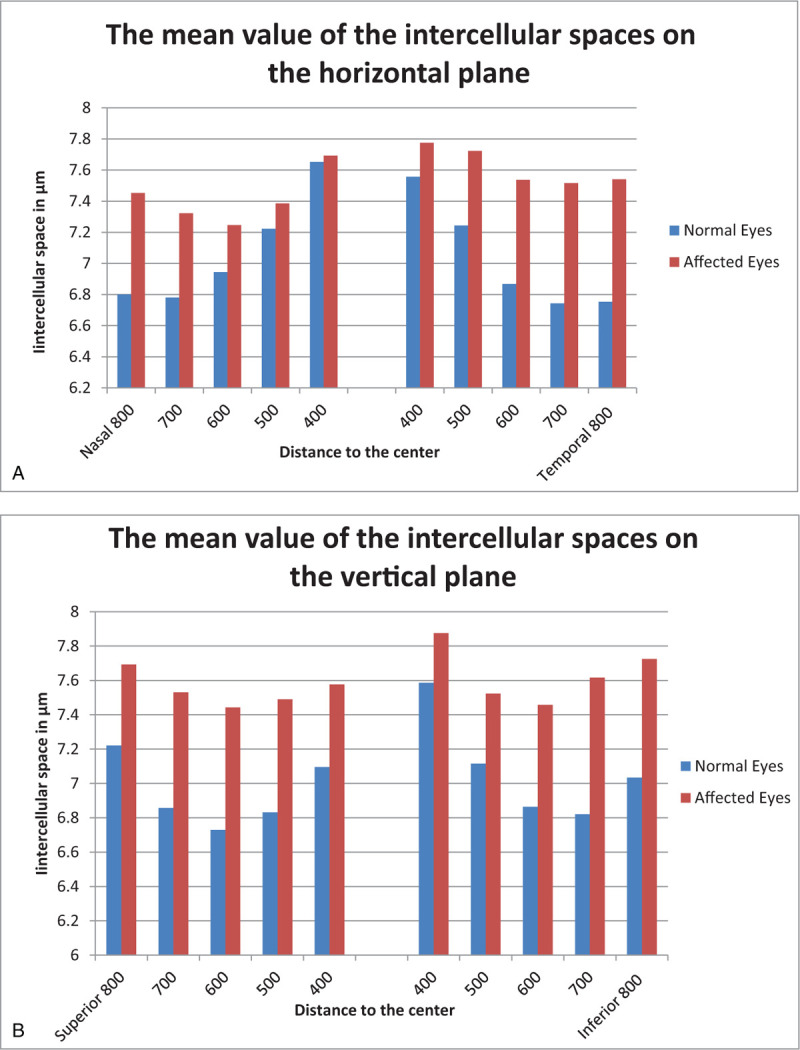
The mean value of the intercellular spaces in healthy and affected eyes. (A) The mean value of the intercellular spaces on the horizontal plane. (B) The mean value of the intercellular spaces on the vertical plane.

The cone densities in healthy eyes (Fig. 3) were considerably higher than those in affected eyes (Fig. 4). Furthermore, the statistical significance of the difference between cone densities in the affected and healthy eyes was greater with increasing distance from the center of the fovea (Table 3). Concerning the IS, they were noticeably larger in the affected eyes (Fig. 5). The smallest IS were around 700 μm in the healthy eyes, and 600 μm in affected ones, on both horizontal and vertical plane. The tendency of the mean of the IS in healthy eyes was to decrease from the center to 700 μm, then tending to increase. A totally different result was observed in the affected eyes, where the trend approached a horizontal line, the mean of the IS being approximately equal at any point of the macula.

**Table 3:**
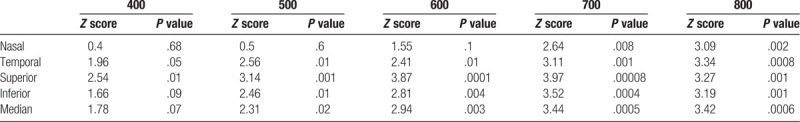
Mann–Whitney *U* test results after comparing the cone densities of healthy and affected eyes.

In all 21 cases, the presence of hypo-AF lesions on the first FAF image correlated with a greater difference between the maximum values of photoreceptor densities in normal and affected eyes (*r* ^2^ = 0.46, *P* = .03). A strong relationship was also detected between the presence of hypo-AF lesions and the duration of the pathology (*r* ^2^ = 0.68, *P* < .001).

## 8 Discussion

Regarding the ChT results, a thicker choroid was found in most cases of CSC compared with healthy eyes. Moreover, detection of a thicker choroid that gradually decreased in size after the disappearance of the SRF indicates the involvement of this anatomical structure in the pathophysiology of CSC.

Photoreceptor density was considerably lower in affected eyes compared with healthy eyes at all distances from the center of the fovea. Due to the inability to detect photoreceptors in the center of the macula (limitation of the resolution of the rtx1 camera^15^,^16^), the trend of cone densities in normal eyes was found to increase from 400 μm to approximately 700 μm and then decrease, as shown in Fig. 3. Figure 4 shows a different trend in eyes with resolved CSC. Notably, the highest and lowest values of cone density in affected and healthy eyes are different. The trend of median values of cone density was more of a straight line in affected eyes, whereas it was a curved line in healthy eyes. The highest, lowest, and median values of cone density in affected eyes seemed to be higher in the nasal and superior quadrants than in the temporal and inferior ones; this could mean that these areas were less affected due to the pathophysiological mechanism of the disease or to gravity.

The values of the IS are perfectly compatible to those of the photoreceptor density, both in healthy eyes and in those with CSC. As shown in Fig. 5, in the healthy eyes, the IS in the temporal and superior quadrants were slightly smaller than the other quadrant in the same plane. However, in affected eyes, like photoreceptor densities, intercellular spaces are slightly smaller in the nasal and upper quadrant. These results only confirm the fact that CSC causes the destruction of the photoreceptors and the increase of the IS.

In addition to the higher maximum density of photoreceptors and smaller IS in healthy eyes, the distributions of these values were different. In healthy eyes, maximum values were found at 800 to 700 μm on the horizontal plane and at 600 to 700 μm on the vertical one. In eyes with resolved CSC, the maximum photoreceptor density values seem to be found closer to the center; 600 μm on the horizontal plane, and 500 to 600 μm on the vertical plane. This could suggest changes in both the number and structure of the photoreceptors, especially in those closer to the center of the fovea.

The lesions found on the FAF images correlated very well with those on the cone mosaic. The hypo-AF regions that were dark on AO images appear to be altered photoreceptors or disruptions of the IZ (as some studies argue).^[8]^ The significance of the Hyper-AF lesions is still difficult to appreciate. At their level, we still see a regular mosaic of photoreceptors, which means that the evolution of these lesions still has to be tracked over time. The laser scars should be observed over time as well. Due to the laser parameters used in CSC, the alteration of these lesions may be different compared with those present in other pathologies.

The strong and positive relationship between FAF and cone density indicates that more hypo-AF lesions on FAF were representative of greater photoreceptor damage and lower photoreceptor density. In addition, the strong correlation of hypo-AF lesions with the duration of pathology indicates that a longer disease duration leads to greater photoreceptor deterioration. Regarding therapeutic solutions, laser therapy allowed a rapid remission of CSC in most cases, thus preventing an even more significant destruction of photoreceptors.

In this study, we investigated the role of laser therapy in patients with CSC who had very good BCVA. In the future, we aim to evaluate patients with lower BCVA, in whom photoreceptors experience greater stress. Another study that could improve the treatment of CSC is comparison of different laser therapy techniques using FAF and AO images.

This study has several limitations, some of them related to the disease itself and others to adaptive optics. One of them was the difficulty to exclude the presence of CSC in the fellow eye in some situations. Changes in the FAF images and especially of AO were present in both eyes in a large proportion of patients. These changes could be CSC sequels, which the patient did not perceive clinically. The above chapter specifies how the CSC diagnosis was excluded and how it was confirmed. If minor changes in the FAF images were present in the fellow eye, care was taken that they preserved the center of the macula. Concerning AO, it was not yet possible to obtain a qualitative mosaic at less than 400 μm from the center. Patients with medium or high hyperopia were excluded from the study because in these patients it was not possible to obtain clear images.^[17]^ Even if the automatic calculation of cone density becomes more and more accurate, in some cases, a small manual correction was still needed.^18^,^19^ Not last in importance, in order to obtain reliable results, this technique required a lot of time for the image acquisition and especially for the image analysis.

In conclusion, although CSC is considered a relatively benign condition that resolves spontaneously in most cases, it causes a significant decrease in photoreceptor density, even in patients with a very good visual acuity. CSC is part of the pachychoroid spectrum of diseases. The presence of hypo-AF lesions and the duration of the pathology are negative prognostic factors of CSC. Laser treatment can reduce the healing period of CSC, thus preventing the loss of photoreceptors. Thus, in daily clinical ophthalmology practice, laser should be the first therapeutic solution considered for patients with CSC, particularly those with hypo-AF lesions in FAF and those with a long-term pathology.

## Author contributions

**Conceptualization:** Radu Ochinciuc, Florian Baltă.

**Data curation:** Radu Ochinciuc, Uliana Ochinciuc.

**Funding acquisition:** Florian Baltă.

**Investigation:** Radu Ochinciuc.

**Methodology:** Ramona Barac.

**Project administration:** Florian Baltă, Marian Burcea.

**Resources:** Florian Baltă.

**Software:** Diana Darabus, Marius Şuţă.

**Supervision:** Marian Burcea.

**Formal analysis:** Horia T. Stanca.

**Visualization:** Horia T. Stanca.

**Writing – original draft:** Radu Ochinciuc.

**Writing – review & editing:** Uliana Ochinciuc, Ramona Barac.
